# Mental Health Burden of German Cancer Patients before and after the Outbreak of COVID-19: Predictors of Mental Health Impairment

**DOI:** 10.3390/ijerph18052318

**Published:** 2021-02-26

**Authors:** Alexander Bäuerle, Venja Musche, Kira Schmidt, Adam Schweda, Madeleine Fink, Benjamin Weismüller, Hannah Kohler, Ken Herrmann, Mitra Tewes, Dirk Schadendorf, Eva-Maria Skoda, Martin Teufel

**Affiliations:** 1 Clinic for Psychosomatic Medicine and Psychotherapy, LVR-University Hospital Essen, University of Duisburg-Essen, 45147 Essen, Germany; venja.musche@uni-due.de (V.M.); kira.schmidt@lvr.de (K.S.); adam.schweda@uni-due.de (A.S.); madeleine.fink@uni-due.de (M.F.); BenjaminMaurice.Weismueller@lvr.de (B.W.); hannah.kohler@uni-due.de (H.K.); Eva-maria.Skoda@uni-due.de (E.-M.S.); martin.teufel@uni-due.de (M.T.); 2 West German Cancer Center, University Hospital Essen, 45147 Essen, Germany; ken.Herrmann@uk-essen.de (K.H.); mitra.tewes@uk-essen.de (M.T.); Dirk.Schadendorf@uk-essen.de (D.S.); 3 Department of Nuclear Medicine, University Hospital Essen, 45147 Essen, Germany; 4 Department of Medical Oncology, University Hospital Essen, 45147 Essen, Germany; 5 Department of Dermatology, University Hospital Essen, 45147 Essen, Germany

**Keywords:** COVID-19, mental health, cancer, anxiety, depression, distress, predictors of mental health impairment

## Abstract

The aim of this study was to analyze individual changes in cancer patients’ mental health before and after the COVID-19 outbreak, and to explore predictors of mental health impairment. Over a two-week period (16–30 March 2020), 150 cancer patients in Germany participated in this study. Validated instruments assessed demographic and medical data, depression and anxiety symptoms (PHQ-2, GAD-2), distress (DT), and health status (EQ-5D-3L). All instruments were adapted to measure the individual mental health before the COVID-19 outbreak. COVID-19-related fear, trust in governmental actions to face COVID-19, and the subjective level of information regarding COVID-19 were measured. Cancer patients showed a significant increase in depression and anxiety symptoms and distress, while health status deteriorated since the COVID-19 outbreak. Increased depression and generalized anxiety symptoms were predicted by COVID-19-related fear. Trust in governmental actions to face COVID-19 and COVID-19-related fear predicted increases in distress. Higher subjective levels of information predicted less increasing anxiety symptoms and distress. Present data suggests that cancer patients experienced a significant increase in mental health burden since the COVID-19 outbreak. Observed predictors of mental health impairment and protective factors should be addressed, and appropriate interventions established, to maintain mental health of cancer patients during the pandemic.

## 1. Introduction

The emergence of the coronavirus disease 2019 (COVID-19), elicited by the severe acute respiratory syndrome coronavirus 2 (SARS-CoV-2), has led to a global public health crisis. On 11 March 2020, the World Health Organization (WHO) officially declared the spread of the virus as a pandemic, the first since H1N1 in 2009/2010 [1]. The pandemic’s magnitude is continuing to rise with over 83,322,449 confirmed infections and 1,831,412 confirmed deaths (as of 3 January 2021) [2]. The lack of vaccinations, up until the end of 2020, and medications to combat the virus effectively has forced governments in most countries to implement various restrictions on public life and limitations on personal freedom to prevent the spread of the virus. These restrictions and limitations on everyday life cause uncertainty and fear and, therefore, affect people’s mental health during this pandemic. 

In fact, a study from Germany has observed that people assess their mental health as significantly worse than before the outbreak of COVID-19 [3]. Depression symptoms, generalized anxiety symptoms, and psychological distress has increased since the outbreak of COVID-19, while individual health status has decreased. In addition to that, recently published international literature showed that mental health burden is a serious issue during the COVID-19 pandemic. Elevated prevalence of distress, depression, and anxiety symptoms were observed in various studies [4,5,6,7,8,9,10,11]. Thereby, being female, at younger age, student or unemployed, a psychiatric history, or experiencing a greater negative impact on the quality of life pose risk factors for increased depression and anxiety symptoms [12,13]. Moreover, psychological burden during this pandemic seemed to persist over a longer course of time. In Germany, COVID-19-related fear decreased within six weeks to the level before the shutdown, while anxiety symptoms and sleep disturbances remained elevated [14]. This study corroborates findings that hint toward the hypotheses that COVID-19-related fear and generalized anxiety symptoms are two discriminant constructs [15]. Data assessed in a longitudinal study conducted shortly after the virus outbreak in China showed that mental health burden persisted for up to one month after the outbreak [16].

People with a pre-existing mental illness appear to be even more burdened during the pandemic due to their increased susceptibility to stress [17,18]. The mental health is not only more affected by COVID-19 in people with pre-existing mental disorders [17,18], but also in patients with pre-existing medical conditions [19], since these conditions lead to a more severe course of COVID-19 [20]. One of the comorbid conditions elevating the risk of a more severe course of COVID-19 is cancer. The immunocompromised status of cancer patients, due to immunosuppressive agents and chemotherapy, increases the risk of infection and worse prognosis [21]. Therefore, this patient group is more susceptible to severe symptoms and suffers from increased mortality. A nationwide analysis (2020) investigated 1590 cases of COVID-19 and found a higher percentage of cancer patients infected with SARS-CoV-2 than in the general Chinese population (based on the report of cancer epidemiology) [22]. This suggests that cancer patients are at higher risk to contract a COVID-19 infection. The results of a different study support the finding of high vulnerability of cancer patients due to COVID-19 as well [23]. Moreover, the course of COVID-19 has been found to be more severe when patients were older and had a higher number of comorbidities [16].

Apart from an objectively increased risk, recent literature on COVID-19 suggests that the vulnerability of cancer patients then, again, leads to an elevated COVID-19-related fear, which forces them to be more cautious and to show more safety behavior during this pandemic [24]. This adds to their pre-existing psychological burden of the cancer diagnosis [25] in that their psychological state might be strained twice, which in turn synergizes to even more elevated distress in times of COVID-19. In fact, the cancer diagnosis itself elicits acute distress in half of the patients, and one third even meet the criteria for at least one mental disorder [26,27]. Thereby, cancer patients most prominently suffer from depression [28,29,30]. The context of COVID-19, therefore, poses a challenge to cancer patients by adding uncertainty and distress to their pre-existing burden [24,31].

A recent Chinese cross-sectional study identified risk factors for mental health problems during the COVID-19 pandemic by examining 6213 cancer patients [31]. Patients with pre-existing mental disorders, excessive alcohol consumption, a higher frequency of feeling overwhelming psychological pressure from COVID-19, a higher frequency of worrying about cancer management due to COVID-19, and a higher level of fatigue and pain were more likely to show a lower mental health status. However, a higher frequency of receiving COVID-19 information, a satisfying quality of life, and good relationships with family members have been identified as protective factors that lower the risk of mental health problems.

The rapidly growing literature on mental health during the COVID-19 pandemic exemplifies the importance of this topic, but also the lack of unambiguous data. Most data is derived from cross-sectional studies, and very few studies used longitudinal study designs to assess mental health during the pandemic. However, the sudden outbreak of the virus is a clear explanation for this lack of methodologically clean studies. Consequently, there are no existing data comparing individual changes in mental health burden in cancer patients since the outbreak of COVID-19. The purpose of this study is to present an approach in which a sample of 150 cancer patients assesses their current mental health and, retrospectively, their mental health prior to the outbreak of COVID-19, as well as to examine predictors of change. 

## 2. Materials and Methods

### 2.1. Procedure and Participants

A cross-sectional study design was applied. The data was acquired through an online survey over the course of two weeks (16–30 March 2020). The survey was distributed via email to cancer patients of the University Hospital Essen. Eligibility for requirement was age ≥18 years, a diagnosis of cancer, cancer treatment at the University Hospital Essen, a good command of the German language, and internet access for all participants. Informed consent was obtained via an electronic form before the participants were asked to complete the survey. Study participation was anonymous, voluntary, and could be terminated at any time without any negative consequences for the participant. The Ethics Committee of the University Hospital Essen approved the study (19-8834-BO).

### 2.2. Measures

The online survey consisted of items on sociodemographic and medical details, validated instruments, adapted instruments, and self-generated items on COVID-19-specific aspects. See Supplementary Materials 1 for the survey, translated into the English language. Sociodemographic data was gathered including age, gender, education, marital status, occupation, and residential situation. Further, type of cancer, tumor-stage and current treatment, time since diagnosis, and prior mental illness were assessed. Health status was assessed via the visual analogue scale from the European Quality of Life 5 Dimensions 3 Level questionnaire (EQ-5D-3L) [32]. The scale ranges from “0 = the worst health you can imagine” to “100 = the best health you can imagine”. Mental health was measured by the Patient Health Questionnaire-2 (PHQ-2), Generalized Anxiety Disorder-2 (GAD-2), and the Distress Thermometer (DT). The PHQ-2 consists of two items screening for depression symptoms over the past two weeks on a 4-point Likert scale (0 = never to 3 = nearly every day) [33]. A sum score of ≥3 points to the presence of major depression symptoms. The GAD-2 consists of two items screening for generalized anxiety symptoms over the past two weeks (4-point Likert scale, 0 = never to 3 = nearly every day) [34,35]. The German version of the DT, involving one visual analogue scale ranging from “0 = no distress” to “10 = extreme distress” was used to measure experienced distress in the past week [36]. A score of ≤5 points to elevated distress. In addition, participants had to retrospectively assess their mental health and health status. To this end, all validated measurements were adjusted to measure the mental health burden and health status of patients before the outbreak of COVID-19.

Further, on a 7-point Likert scale (1 = very low to 7 = extremely high), participants could rate their *COVID-19-related fear* (I worry about COVID-19). A 7-point Likert scale, ranging from 1 = complete disagreement to 7 = complete agreement, was used to assess *trust in governmental actions to face COVID-19* (I think all governmental measures are being taken to combat COVID-19; I have confidence in the governmental system in Germany; I think Germany is well prepared to face COVID-19), and *the subjective level of information regarding COVID-19* (I feel informed about COVID-19; I understand the health authorities’ advice regarding COVID-19; I feel informed about measures to avoid an infection with COVID-19). Cronbach’s α was used as an indication of internal consistency in order to test the reliability for both scales. Both showed high internal consistency with Cronbach’s *α* = 0.890 and Cronbach’s *α* = 0.829, respectively. Similar *α* could be observed in previous studies [3,4].

### 2.3. Statistical Analyses

Statistical analyses were conducted using the statistical program for social sciences SPSS version 26 (IBM, New York, NY, USA), as well as R (4.0.3). Mean scores for EQ-5D-3L, DT, and COVID-19-related fear, as well as the scales trust in governmental actions and subjective level of information, were calculated. Furthermore, sum scores for PHQ-2 and GAD-2 were calculated. The Kolmogorov–Smirnov test was applied to test for normal distribution of the data. Since the data showed a non-normal distribution, we applied non-parametric tests. Descriptive statistics were performed for sociodemographic data, scores of psychometric measures, and assessment of COVID-19-related scales. Wilcoxon signed rank tests were applied comparing PHQ-2, GAD-2, DT, and EQ-5D-3L before and after the COVID-19 outbreak in cancer patients. We used Cohen’s *d* to report effect sizes, while a *d* value around 0.2, 0.5, and 0.8 is considered as small, medium-sized, and large effect, retrospectively [37]. In order to determine the difference between mental health burden and health status before and after the outbreak of COVID-19, difference values of PHQ-2, GAD-2, DT, and EQ-5D-3L were computed. A multivariate multiple regression analysis was performed to identify possible predictors for the differences in mental health and health status. Pre-post differences in PHQ, GAD, distress, and the EQ-5D-3L were added as dependent variables. In accordance with previous research [3], mental illness, COVID-19-related fear, trust in governmental actions, and subjective level of information of COVID-19 were included as possible predictors. Breusch–Pagan tests indicate homoscedasticity with all *p* > 0.05. Regression residuals are skewed, but linear regression has been shown to yield reliable estimates of coefficients and standard errors under violation of residual normality when the sample sizes are reasonably high [38]. The variance inflation factor (VIF) values indicated no multicollinearity between the predictors with values <10 [39]. The level of significance was set at *α* = 0.05 (two-sided tests).

## 3. Results

### 3.1. Sample Description

Of the 150 cancer patients, 78 were female (52%) and 72 were male (48%). Most participants were aged between 45 and 74 years (81.4%). They reported different types of cancer in various tumor-stages, with a mean of 30.85 months (*SD* = 45.695) since diagnosis (including first diagnosis, recidivism, and metastatic cancer), and 44.7% of the patients reported metastatic cancer. Table 1 includes all sociodemographic and medical data.

### 3.2. Differences in Mental Health and Health Status before and after the Outbreak of COVID-19

**Figure 1:**
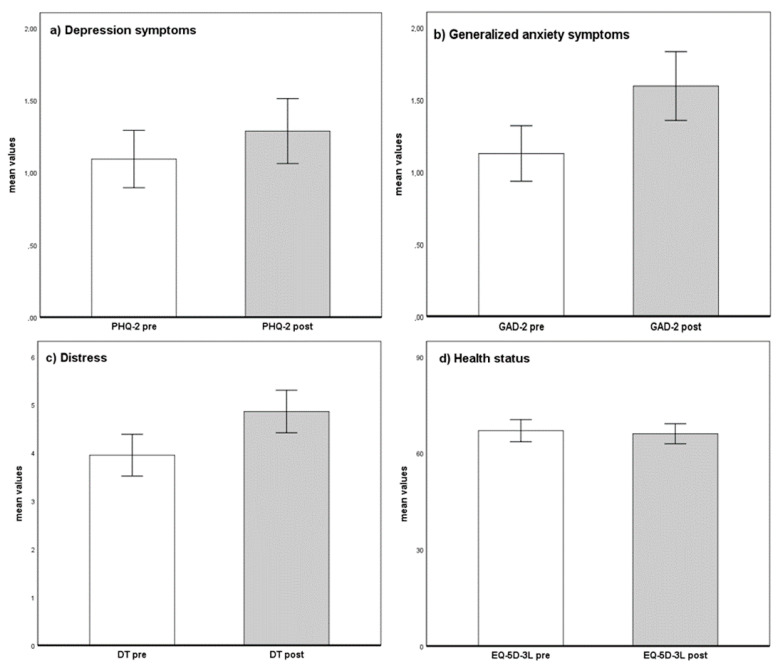
Mental health and health status mean scores before and after the COVID-19 outbreak. Mean values and 95% CI as error bars before (pre) and after (post) the COVID-19 outbreak of PHQ-2: Patient Health Questionnaire-2, M_PHQ-2 pre_ = 0.91 vs. M_PHQ-2 post_ = 1.14 (**a**); GAD-2: Generalized Anxiety Disorder Scale-2, M_GAD-2 pre_ = 1.06 vs. M_GAD-2 post_ = 1.52 (**b**); DT: Distress Thermometer M_DT pre_ = 3.91 vs. M_DT post_ = 4.94 (**c**); EQ-5D-3L: European Quality of Life 5 Dimensions 3 Level, M_EQ-5D-3L pre_ = 82.32 vs. M_EQ-5D-3L post_ = 80.27 (**d**).

The results of the Wilcoxon signed rank test comparing mean values of PHQ-2, GAD-2, DT, and EQ-5D-3L before and after the outbreak of COVID-19 revealed a significant increase in depression symptoms, *Z* = −2.551, *p* = 0.011, *d* = 0.148, generalized anxiety symptoms, *Z* = −5.042, *p* < 0.001, *d* = 0.345, distress, *Z* = −6.763, *p* < 0.001, *d* = 0.336, and a deterioration in health status, *Z* = −2.850, *p* = 0.004, *d* = 0.047. An overview of the mean scores of PHQ-2, GAD-2, DT, and EQ-5D-3L in cancer patients before and after the outbreak of COVID-19 is shown in Figure 1.

### 3.3. Prevalence of Depression Symptoms, Generalized Anxiety Symptoms, and Distress before and after the COVID-19 Outbreak

The prevalence of major depression symptoms increased from 9.3% before to 16.7% after the outbreak of COVID-19, while the prevalence of severe generalized anxiety symptoms elevated from 8.0% to 20.7% after the outbreak. Enhanced distress was reported by 38% of the participants before the outbreak and by 54.7% after the outbreak. For an overview, see Table 2.

### 3.4. Predictors of Change in Mental Health and Health Status

On a global level, multivariate tests indicate that COVID-19-related fear (Pillai’s trace = 0.123, F(4, 142) = 4.97, *p* < 0.001), as well as subjective level of information (Pillai’s Trace = 0.09, F(4, 142) = 3.49, *p* = 0.009), are significantly associated with one or some of the dependent variables. No significant multivariate effects were found for trust in governmental interventions (Pillai’s Trace = 0.035, F(4, 142) = 1.27, *p* = 0.283). The results of the following univariate multiple regression models predicting change in mental health, i.e., depression symptoms (PHQ-2), generalized anxiety symptoms (GAD-2), and distress (DT), and health status, i.e., EQ-5D-3L, are shown in Table 3, Table 4, Table 5 and Table 6. As Table 3, Table 4, Table 5 and Table 6 show, the increase in depression symptoms could be significantly explained by COVID-19-related fear. Mental illness, subjective level of information, and trust in governmental actions were not significant predictors. The model provided 7.2% of explained variance. The change in generalized anxiety symptoms could be significantly explained by COVID-19-related fear and subjective level of information. Again, mental illness and trust in governmental actions were not significant predictors. The explained variance was 13.8%. The change in distress could be significantly explained by COVID-19-related fear, subjective level of information, and trust in governmental actions with an explained variance of 10.5%. In contrast, no predictor could explain the reported change in health status.

## 4. Discussion

This study investigated the changes in mental health and health status of cancer patients since the outbreak of COVID-19, as well as predictors of mental health impairment. The study compared mental health as well as somatic health status before and after the COVID-19 outbreak in cancer patients. Major depression symptoms, as well as severe generalized anxiety symptoms and elevated distress, were more frequent since the outbreak of COVID-19. A small effect was observed when comparing depression symptoms before and after the outbreak. Small to medium-sized effects were observed in terms of increase in generalized anxiety symptoms and distress. The deterioration in health status was significant but actually very small. Further analyses to identified predictors for the increase in mental health burden and decrease in health status were conducted. Elevated COVID-19-related fear predicted an increase in depression and generalized anxiety symptoms, while higher subjective levels of information regarding COVID-19 predicted less increase in generalized anxiety symptoms. Increased distress was predicted by COVID-19-related fear and trust in governmental actions to face COVID-19 (although only in the univariate regressions), while higher subjective level of information predicted less increase in distress. The reported deterioration in cancer patients’ health status since the onset of COVID-19 could not be explained by any predictor. Prior mental illness had no significant effect on the increase in mental health burden or decrease in cancer patients’ health status.

These findings are in line with previous research showing an elevated mental health burden in the general population [4,5,6,7,8,9,10,11] and in cancer patients [24,31]. In fact, one study comparing cancer patients to matched controls observed no significant differences in mental health burden between the two groups [24]. Nevertheless, both groups had increased mental health burden as compared to previous representative validation samples. Furthermore, the pattern of elevated mental health burden observed in this sample of cancer patients since the onset of COVID-19 is similar to the increase of mental health burden in the general population [3]. This highlights the need for support approaches to prevent the manifestation of mental health related problems in cancer patients. Low-threshold and contact-free interventions offer great advantages to support burdened cancer patients in times of social isolation [40]. Existing interventions proved efficient in reducing distress in cancer patients [41,42], while other studies are still in progress [43].

Elevated COVID-19-related fear predicted an increased mental health burden since the outbreak of COVID-19, which is in line with previous results from a study investigating cancer patients in China [31] and a study from Germany investigating the general population [3]. However, not only is increased mental health burden associated with high COVID-19-related fear, but also adherent safety behaviors, such as increased hand hygiene, in the general population [44] and in cancer patients [24]. According to these findings, increased COVID-19-related fear is associated with increased mental health burden on the one hand, and supports adherent safety behaviors on the other, which is important to protect such a vulnerable group from infections. Feeling well-informed about COVID-19 predicted less increase in generalized anxiety symptoms and distress, which is in line with previous research showing that a high subjective level of information was associated with less mental health burden in the general population during the pandemic [4]. Moreover, the feeling of being well-informed about COVID-19 predicted less mental health burden in cancer patients during the pandemic [31] and predicted less increase in mental health burden since the outbreak of COVID-19 in the general population [3]. The importance of understandable and clear information in cancer patients is widely known, as it might be associated with self-efficacy, reduced uncertainty, and mental health burden [45,46,47]. Therefore, authorities should offer low-threshold, easily comprehensible educational resources for cancer and COVID-19 in order to foster a sense of clarity and to prescribe appropriate guidelines for everyday behavior during the pandemic. Prior mental illness was, in contrast to many previous studies, no predictor for an increase in mental health burden [3,17,18,31]. In this context, it is important to mention that only a small proportion of the sample reported a mental illness. Thus, the effect could have not been detectable due to the small group size. Further, patients with a mental illness might have already reported a high mental health burden and it increased not as much as for patients without a mental illness.

When interpreting the data, limitations need to be considered. As a cross-sectional study design was applied, causality cannot be assumed. Existing validated instruments were adapted to the research question, as no instruments to assess mental health prior to the onset of COVID-19 exists. Therefore, no validated instruments were used to assess mental health before the outbreak of COVID-19. Rather, validated instruments were adjusted to assess mental health before the COVID-19 outbreak retrospectively. Imitated representatively of the enrolled cancer sample is evident. Non-responders could not be identified due to the anonymous approach of data assessment. Thus, the possibility of selection bias should be taken into account. Previous studies have shown that mental health and COVID-19-related fear seem to vary over time [14,48]. Thus, the time of study participation might have an impact on the self-reported data. Since this study was conducted during an early stage of the pandemic in Germany, it would be important to investigate the influence of the pandemic on mental health of cancer patients at a different stage of the pandemic. In this study, we only examined whether a mental illness had been previously diagnosed, not the type of mental illness. This is an important limitation, because the spectrum of mental illness is broad. Furthermore, it is important to consider that many participants could not state their tumor stage. This might be an important parameter affecting the distress as well as quality of life in cancer patients, as in previous research the psychological burden has been shown to vary between different tumor stages [49,50]. Last, the reasons for an increase in mental health burden could be broad and varied, e.g., social compartmentalization and the obligation to wear a mask, but also exacerbation of the cancer illness. It is therefore important to not consider all measured effects as caused by COVID-19. The occurrence of recall-biased assessments should be considered, since the mental health before the outbreak of COVID-19 was retrospectively assessed. Nevertheless, no study exists showing longitudinal data in terms of mental health in cancer patients before and after the outbreak of COVID-19. Therefore, the approach applied in this study is a practicable way to pursue the study’s goal.

## 5. Conclusions

In conclusion, cancer patients enrolled in this study reported an increase in mental health burden and deterioration in somatic health status since the COVID-19 outbreak. Elevated COVID-19-related fear predicted the increase in mental health burden, while prior mental illness was no predictive factor. The data suggested that a high level of information regarding COVID-19 had a protective effect in terms of change in generalized anxiety symptoms and distress. Innovative and contact-free interventions are needed to support cancer patients in times of social distancing to maintain mental health. Furthermore, it is necessary that authorities and healthcare providers establish and offer adequate cancer-specific information regarding COVID-19.

## Figures and Tables

**Table 1:** Sociodemographic and medical characteristics.

	N	%
**Sex**		
Female	78	52.0
Male	72	48.0
**Age**		
<45 years	17	11.3
45–74 years	122	81.4
≥75 years	11	7.3
**Marital status**		
Single	12	8.0
Married	110	73.3
In a relationship	15	10.0
Divorced/separated	7	4.7
Widowed	6	4.0
**Educational level**		
University education	50	33.3
Higher education entrance qualification	45	30.0
Higher secondary education	39	26.0
Lower secondary education	16	10.7
**Employment**		
Full employment	35	23.3
Partial employment	12	8.0
Not employed	11	7.3
Retirement	55	36.7
Sick leave	22	14.7
Other	15	10.0
**Community size (Population)**		
100,000 residents	82	54.7
20,000 residents	43	28.7
5000 residents	16	10.7
<5000 residents	9	6.0
**Mental illness**		
yes	15	10.0
no	135	90.0
**Tumor Stage**		
I	10	6.7
II	11	7.3
III	21	14.0
IV	36	24.0
Unknown	72	48.0
**Treatment situation**		
Curative	24	16.0
Palliative	24	16.0
Aftercare	37	24.7
Could not be assessed	30	20.0
Currently not decidable	35	23.3
**Type of cancer**		
Skin cancer	36	24.0
Cancer of the gastrointestinal tract	23	15.3
Lung cancer	17	11.3
Urogenital cancer	13	8.7
Thyroid cancer	12	8.0
Bone cancer, cartilage tumor, soft-tissue sarcoma	11	7.3
Head and neck cancer	10	6.7
Breast cancer	8	5.3
Leukemia or Lymphoma	7	4.7
Cancer of the central nervous system	7	4.7
Cancer of the eye	6	4.0
**Total**	150	100

**Table 2:** Prevalence of depression symptoms, generalized anxiety symptoms, and distress before and after the outbreak of COVID-19 in cancer patients.

	Before COVID-19 Outbreak	After COVID-19 Outbreak
PHQ-2		
<3	136 (90.7%)	125 (83.3%)
≥3	14 (9.3%)	25 (16.7%)
GAD-2		
<3	138 (92.0%)	119 (79.3%)
≥3	12 (8.0%)	31 (20.7%)
DT		
<5	93 (62%)	68 (45.2%)
≥5	57 (38%)	82 (54.7%)
Total	150 (100%)	150 (100%)

Note: PHQ-2 = Patient Health Questionnaire-2, a sum score of ≥3 indicates major depression symptoms; GAD-2 = Generalized Anxiety Disorder Scale-2, a sum score of ≥3 indicates severe generalized anxiety symptoms; DT = Distress Thermometer, a score of ≥5 indicates elevated distress in oncological patients.

**Table 3:** Regression coefficients predicting an increase in depression symptoms (PHQ-2).

	*b*a	*β*	*SE*a	*t*-Value	*p*-Value
Intercept	0.083		0.641	0.13	0.897
Mental illness	0.204	0.066	0.249	0.819	0.414
COVID-19-related fear	0.138	0.236	0.047	2.928	0.004
Trust in government	0.002	0.003	0.068	0.028	0.977
Subjective level of information	−0.103	−0.087	0.11	−0.938	0.35

*Note*. PHQ-2 (difference between before and after the COVID-19 outbreak) = dependent variable, *R*^2^ = 0.072, *F*(4) = 2.819, *p* = 0.027, *n* = 150. a: Unstandardized regression coefficients.

**Table 4:** Regression coefficients predicting an increase in generalized anxiety symptoms (GAD-2).

	*b*a	*β*	*SE*a	*t*-Value	*p*-Value
Intercept	0.915		0.691	1.325	0.187
Mental illness	0.145	0.042	0.268	0.541	0.59
COVID-19-related fear	0.199	0.304	0.051	3.92	<0.001
Trust in government	0.072	0.089	0.073	0.987	0.325
Subjective level of information	−0.303	−0.228	0.118	−2.566	0.011

Note. GAD-2 (difference between before and after the COVID-19 outbreak) = dependent variable, *R*^2^ = 0.138, *F*(4) = 5.814, *p* < 0.001, *n* = 150. a: Unstandardized regression coefficients.

**Table 5:** Regression coefficients predicting an increase in distress (DT).

	*b*a	*β*	*SE*a	*t*-Value	*p*-Value
Intercept	2.25		1.066	2.111	0.036
Mental illness	0.258	0.05	0.413	0.625	0.533
COVID-19-related fear	0.188	0.19	0.078	2.405	0.017
Trust in government	0.234	0.188	0.113	2.07	0.04
Subjective level of information	−0.577	−0.288	0.182	-3.167	0.002

Note. DT (difference between before and after the COVID-19 outbreak) = dependent variable, *R*^2^ = 0.105, *F*(4) = 4.237, *p* = 0.003, *n* = 150. a: Unstandardized regression coefficients.

**Table 6:** Regression coefficients predicting a deterioration in health status (EQ-5D-3L).

	*b*a	*β*	*SE*a	*t*-Value	*p*-Value
Intercept	−5.238		9.649	−0.543	0.588
Mental illness	−4.806	−0.107	3.742	−1.284	0.201
COVID-19-related fear	−0.365	−0.043	0.708	−0.516	0.607
Trust in government	0.235	0.022	1.025	0.229	0.819
Subjective level of information	0.887	0.051	1.648	0.538	0.591

Note. EQ-5D-3L (difference between before and after the COVID-19 outbreak) = dependent variable, *R*^2^ = 0.020, *F*(4) = 0.740, *p* = 0.566, *n* = 150. a: Unstandardized regression coefficients.

## Data Availability

The raw data supporting the conclusions of this article will be made available by the corresponding author, on request.

## Supplementary Materials

The following are available online at https://www.mdpi.com/1660-4601/18/5/2318/s1, Supplementary Materials 1 for the survey, translated into the English language.
