# Supplementary material for: Mental Health Burden of German Cancer Patients before and after the Outbreak of COVID-19: Predictors of Mental Health Impairment
Source: Int J Environ Res Public Health. 2021 Feb 26;18(5):2318. doi: 10.3390/ijerph18052318 (PMC7967708; doi:10.3390/ijerph18052318)
Supplement: Supplementary file 1 [file ijerph-18-02318-s001.pdf]

Supplementary Material 1

Full questionnaire translated from German into English language.

Sociodemographic Questions

Please indicate your gender.

Please indicate which age category you fall into.

Please indicate your current family status.

Please indicate your highest level of education.

Please indicate which employment status applies to you.

Please indicate what type of city or town you live in.

Medical Information

Please name your cancer disease.

Please indicate the stage of your disease (tumor stage).

Please indicate your current treatment situation.

Please indicate the date of diagnosis

COVID-19-specific Items

Trust in governmental actions

I think Germany is well prepared to face COVID-19.

I think all governmental measures are being taken to combat COVID-19.

I have confidence in the governmental system in Germany.

Subjective level of information about COVID-19

I feel informed about COVID-19.

I feel informed about measures to avoid an infection with COVID-19.

I understand the public health authorities‘ advices regarding COVID-19.

COVID-19-related fear

I worry about COVID-19.

Validated measures

Patient-Health-Questionnaire 2 (PHQ-2)

Over the last 2 two weeks, how often have you been bothered by the following problems?

1. Little interest or pleasure in doing things
2. Feeling down, depressed or hopeless

Generalized Anxiety Disorder Scale-2 (GAD-2)

Over the past 2 weeks, how often have you been bothered by any of the following problems?

1. Feeling nervous, anxious or on edge
2. Not being able to stop or control worrying

Distress Thermometer (DT)

Please indicate on the slider the number (0-10) that best describes how stressed you have felt in the last week, including today.

Visual analogue scale of the European Quality of Life 5 Dimensions 3 Level Questionnaire (EQ-5D-3L)

Please enter the number on the slider that best reflects your current health status.

Validated measures adjusted

Patient-Health-Questionnaire-2 (PHQ-2) – adjusted

How often did you feel affected by the following complaints before the outbreak of COVID-19 (corona virus)?

1. Little interest or pleasure in doing things
2. Feeling down, depressed or hopeless

Generalized Anxiety Disorder Scale-2 (GAD-2) -adjusted

How often did you feel affected by the following complaints before the outbreak of COVID-19 (corona virus)?

1. Feeling nervous, anxious or on edge
2. Not being able to stop or control worrying

Distress Thermometer - adjusted

Please indicate on the slider the number (0-10) that best describes how burdened you felt prior to the COVID-19 (Corona virus) outbreak.

Visual analogue scale of the European Quality of Life 5 Dimensions 3 Level Questionnaire (EQ-5D-3L) - adjusted

Please indicate on the slider the number that best reflects your health status prior to the COVID-19 (corona virus) outbreak.
